# Microwave Assisted Synthesis of Novel Functionalized Hydantoin Derivatives and Their Conversion to 5-(*Z*) Arylidene-4*H*-imidazoles

**DOI:** 10.3390/molecules16075527

**Published:** 2011-06-29

**Authors:** Sukanta Kamila, Haribabu Ankati, Edward R. Biehl

**Affiliations:** Department of Chemistry, Southern Methodist University, 3215 Daniel Avenue, Dallas, TX 75275, USA

**Keywords:** MW assisted synthesis, hydantoin, arylidene-4*H*-imidazoles

## Abstract

2-(Alkyl-1-yl)-1*H*-imidazol-5(4*H)*-ones **5a–n** were synthesized via nucleophilic substitution of the methylsulfanyl group of the corresponding 2-(methylthio)-1*H*-imidazol-5(4*H*)-ones **3a–c** with suitably substituted secondary amines. The starting 2-thioxo- imidazolidin-4-ones **2a**,**2b** were prepared by condensation of thiohydantoin and benzo[*b*]-thiophene-3-carbaldehyde or benzofuran-3-carbaldehyde under microwave irracdiation (MW) conditions. 2-Methylthio derivatives **3a–c** were prepared by treatment of **2a–b** with methyl iodide in the presence of aqueous sodium hydroxide.

## 1. Introduction

**Figure 1:**
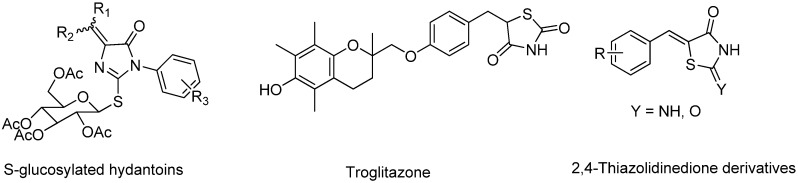
Chemical structure of several biologically important compounds.

Hydantoin derivatives have achieved considerable success as anticonvulsant agents [1]. The nucleosides of several 5-arylidene-3-arylhydantoins and 2-thiohydantoins show potent activity against human immunodeficiency virus (HIV) [2] and the leukemia subpanel [3]. A recent study showed that *S*-glucosylated hydantoins (Figure 1) act against herpes simplex virus, type 1 (HSV-1) and type 2 (HSV-2) in Vero cells [4]. A further study reported that thiazolidinediones (TZDs), which are known to have potent enhancing effects on insulin sensitivity, have been developed for the treatment of noninsulin-dependent diabetes mellitus [5,6].

It also been found that TZDs are high-affinity ligands for peroxisome proliferitor-activated receptor-γ (PPAR-γ) and inhibit the production of monocyte chemo attractant protein 1 (MCP-1) in some human tissues [6]. Another group of TZDs revealed that troglitazone (Figure 1) acts on acute liver injury induced by ethanol and lipopolysaccharide [7]. For the past few years our group has been working on biologically important compounds [8,9,10]. Herein we report on the synthesis of variety of benzo[*b*]thiophene and benzofuran based hydantoin derivatives with the aim of investigating their antimicrobial and neuroprotecting properties.

## 2. Results and Discussion

**Scheme 1:**
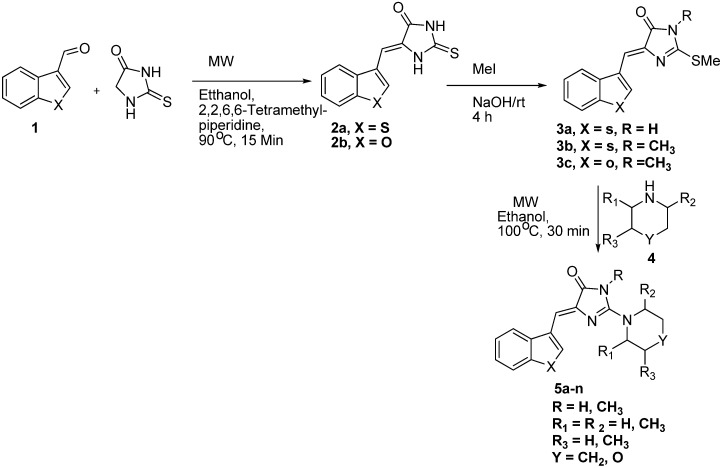
Schematic representation for the synthesis of compounds **5a–n**.

As shown in Scheme 1, various substituted hydantion derivatives **5a–n** were prepared by the condensation between benzo[*b*]thiophene-3-carbaldehyde and benzofuran-3-carbaldehyde with thiohydantoin using MW irradiation and a catalytic amount of 2,2,6,6-tetramethyl piperidine in ethanol that yielded (*Z*)-5-(benzo[*b*]thiophen-3-ylmethylene)-2-thioxoimidazolidin-4-one (**2a**) and (*Z*)-5-(benzofuran-3-ylmethylene)-2-thioxoimidazolidin-4-one (**2b**), respectively.

Since the starting material for **2b** is not commercially available, it was prepared according to the literature procedure [11,12] which involved converting 2-hydroxy acetophenone to 3-methylbenzofuran [11] which was subsequently transformed to benzofuran-3-carbaldehyde by refluxing with SeO_2_ in 1,4-dioxane [12].

Although many bases shown in Table 1 can be used as catalysts [e.g., piperidine, pyridine, *N*-methylpiperidine (NMP), DBU)], 2,2,6,6-Tetramethylpiperidine (TMP) works best. The same reaction under conventional reflux condition using ethanol as solvent gave lower yields after longer times (5 h) and/or compounds that required rigorous purification. However the MW reaction provides cleaner reactions, shorter times (15 min) and purification of the products only required washing with cold ethanol. In addition the yields are good to excellent. The optimum temperature and condition for this MW assisted reaction was determined by a series of reactions of appropriate aldehyde **1** with hydantoin. The results are summarized in Table 1. The results indicate that MW irradiation at 90 °C for 15 min in ethanol is the optimum condition for the synthesis of **2a** and **2b**.

**Table 1:** Screening of solvents, reaction time and temperature for the synthesis of **2a**.

Entry	Base	Condition ^a^	Temp. (°C)	Time (min)	Yield (%) ^b^
1	-	No solvent	90	15	trace
2	-	Ethanoll	90	15	trace
3	Piperidine	Ethanol	90	15	80
4	TMP	Ethanol	90	15	96
5	TMP	No solvent	90	15	trace
6	DBU/Pyridine	Ethanol	90	20	20
7	TMP	Acetonitrile	90	15	76
8	TMP	Acetonitrile	130	15	15
9	DBU	Acetonitrile	90	30	trace
10	TMP	DMF	90	15	45
11	NMP	DMF	90	30	10
12	DBU	DMF	120–140	15	trace
13	TMP	Water	90	15	trace
14	TMP	Water	130	15	trace
15	-	water	130	30	trace
16	TMP	Tolune	90	15	trace
17	TMP	Isopropanol	90	15	45
18	TMP	THF	90	15	38
19	TMP	n-Butanol	90	15	33

^a^ All the reaction was carried out in equimolar amount of each compound in 2 mL of solvent at 150 psi pressure; ^b^ Isolated yield.

The *Z* configuration of the compounds **2a** and **2b** was confirmed by comparison of previously reported [2] hydantoin derivatives along with nuclear Overhauser effect (NOE) experiments [4]. The vinylic proton in the ^1^H-NMR spectra of benzo[*b*]thiophene and benzofuran derivatives appeared as singlets at 6.72 ppm and 6.58 ppm, respectively. The intermediates **2a** and **2b** were in turn transformed into the corresponding methylsulfanyl derivatives **3**(**a–c**) by alkylation with methyl iodide in basic medium [13]. The use of 1.3 equiv. of alkyl halide and stirring for 4 h afforded **3a** while use of excess alkylating agent (2.5 equiv.) and stirring for 16 h afforded dialkylation giving **3b** in high yield. The ^13^C-NMR spectrum of compounds **3a–c** showed the absence of C=S signal of **2a**, **2b** at 179.50 ppm and the appearance of the C=N signal at 170.9 ppm, corresponding to s-alkylation. On the other hand, the ^1^H-NMR spectra of **2a** and **2b** showed two NH signals at 12.41 and 12.18 which were absent in the spectra of **3a** and **3b**, in which only a broad peak NH signal at 11.84 ppm appeared. Conversion of **2a** to **3a** via microwave irradiation was unsuccessful. The intermediate methylsulfanyl derivatives were subsequently converted into compounds **5a–n** by nucleophilic substitution of the methylsulfanyl group with suitably substituted secondary amines [14] by using MW irradiation of a solution containing excess amine (>12.5 equiv.) and absolute ethanol at an elevated temperature (100 °C). The same reaction under conventional reflux condition using ethanol as solvent required longer time (16 h) and/or rigorous purification of products As shown in Table 2, the 5-(*Z*) arylidene-4*H*-imidazole compounds **5a–n** were formed in good to excellent yields. In most cases, the 5-(*Z*) arylidene-4*H*-imidazoles precipitated upon cooling the reaction mixture and only simple trituration with ethanol-hexane followed by recrystallization from ethanol afforded the product **5a–l**. But in case of **5m** and **5n**, the crude reaction mixtures were purified by column chromatography using 40% ethyl acetate-hexane mixture (v/v). All the products were well characterized by ^1^H-NMR, ^13^C-NMR, IR and HRMS.

**Table 2:** MW assisted synthesis of various 5-(*Z*) arylidene-4*H*-imidazoles.

Entry	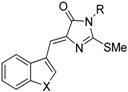	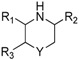	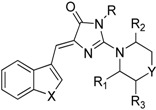	% Yield ^a,b^
1	X = S, R = H	R_1_ = R_2_ = R_3_ = H, Y = CH_2_	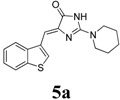	91
2	X = S, R = H	R_1_ = R_2_ = R_3_ = H, Y = O	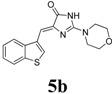	90
3	X = S, R = H	R_1_ = R_2_ = H, R_3_ = CH_3_, Y = CH_2_	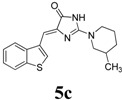	86
4	X = S, R = CH_3_	R_1_ = R_2_ = R_3_ = H, Y = CH_2_	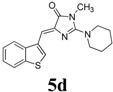	89
5	X = S, R = H	R_1_ = R_2_ = CH_3_, R_3_ = H, Y = O	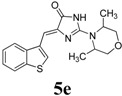	80
6	X = S, R = CH_3_	R_1_ = R_2_ = R_3_ = H, Y = O	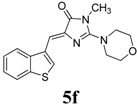	92
7	X = S, R = CH_3_	R_1_ = R_2_ = H, R_3_ = CH_3_, Y = CH_2_	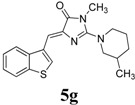	88
8	X = S, R = H	R_1_ = R_2_ = R_3_ = H, Y = S	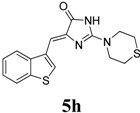	85
9	X = S, R = H	R_1_ = R_2_ = R_3_ = H, Y = NCH_3_	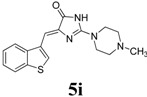	86
10	X = O, R = CH_3_	R_1_ = R_2_ = R_3_ = H, Y = CH_2_	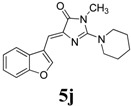	86
11	X = O, R = CH_3_	R_1_ = R_2_ = R_3_ = H, Y = O	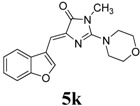	91
12	X = O, R = CH_3_	R_1_ = R_2_ = R_3_ = H, Y = S	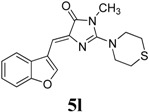	90
13	X = O, R = CH_3_	R_1_ = R_2_ = R_3_ = H, Y = NCH_3_	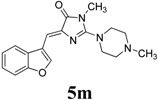	88
14	X = O, R = CH_3_	R_1_ = R_2_ = CH_3_, R_3_ = H, Y = O	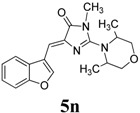	77

^a^ Isolated yield; ^b^ All the products were characterized by IR, ^1^H-NMR, ^13^C-NMR, DEPT, HRMS and elemental analysis.

The use of aromatic amines for the same reaction was unsuccessful. From Table 1, it can be seen that in the case of 3,5-dimethylmorpholine (entry 14) the comparatively low yield probably reflects unfavorable steric interaction between the two methyl groups.

## 3. Experimental

### General

The ^1^H- and ^13^C-NMR spectra were recorded on a 500-MHz Jeol multinuclear NMR spectrometer; chemical shifts were referenced to tetramethylsilane (TMS) as internal standard. Infrared (IR) spectra were obtained on a Varian 3100 Fourier transform (FT)-IR Spectrometer. Melting points were taken on a Meltemp apparatus. All chemicals and reagents were purchased from commercial sources. Mass spectra were obtained from Washington University, St. Louis. MO. Microwave experiments were carried out on CEM Discover microwave instrument.

*(Z)-5-(Benzo[b]thiophen-3-ylmethylene)-2-thioxoimidazolidin-4-one* (**2a**) *under MW irradiation*. An equimolar quantity of benzo[*b*]thiophene-3-carbaldehyde and thiohydantoin were mixed in a mortar pestle and charged into a specially designed MW test tube. After the addition of ethanol (2 mL) followed by 1–2 drops of 2,2,6,6-tetramethylpiperidine, the test tube was sealed then irradiated for 15 min at 90 °C and 150 psi pressure. After cooling, the solid mass was scraped out of the test tube and placed into a flask containing 95% ethanol (20 mL) and filtered. The solid mass was collected then washed with ethanol (20 mL) and dried under reduced pressure and the residue was recrystallized from ethanol to afford 0.49 g (96%) of (*Z*)-5-(benzo[*b*]thiophen-3-ylmethylene)-2-thioxoimidazolidin-4-one (**2a**) as yellow floppy crystals. m.p.: 259–261 °C; IR (KBr) ν (cm^−1^): 3274, 1723, 1649, 1496; ^1^H-NMR (DMSO-*d_6_*): δ: 12.41 (brs, 1H, NH), 12.18 (brs, 1H, NH), 8.49 (s, 1H, Ar–CH), 8.02 (dd, *J* = 7.45 Hz, 8.00 Hz, 2H, Ar–CH), 7.46–7.42 (m, 2H, Ar–CH), 6.72 (s, 1H, vinylic proton); ^13^C-NMR (DMSO-d_6_) δ: 179.5 (C), 166.1 (C), 139.1 (C), 138.4 (C), 129.9 (CH), 129.0(C), 127.8 (C), 125.7 (CH), 125.6 (CH), 123.6 (C), 122.0 (CH), 102.3 (CH); HRMS *m/z*: 261.0161 found (calculated for C_12_H_8_N_2_O_2_S_2_, [M+H]^+^ requires 261.0158).

*(Z)-5-(Benzofuran-3-ylmethylene)-2-thioxoimidazolidin-4-one* (**2b**). Following the procedure for the preparation of **2a**, the microwave-assisted reaction of equimolar quantitites of benzofuran-3-carbaldehyde and thiohydantoin gave 0.51 g (91%) of **2b** as a dark brown solid. m.p.: 307–308 °C; IR (KBr) ν (cm^−1^): 3209, 1725, 1648, 1484, 1452; ^1^H-NMR (DMSO-*d_6_*) δ: 12.39 (brs, 1H, NH), 11.95 (brs, 1H, NH), 8.83 (s, 1H, Ar–CH), 7.88 (d, *J* = 8 Hz, 1H, Ar–CH), 7.61 (d, *J* = 8 Hz, 1H, Ar–CH), 7.38–7.30 (m, 2H, Ar–CH), 6.58 (s, 1H, vinylic proton); ^13^C-NMR (DMSO-*d_6_*) δ: 179.2 (C), 165.7 (C), 154.7 (C), 147.1 (CH), 128.7 (C), 126.6 (C), 125.9 (CH), 124.1(CH), 120.3 (CH), 114.3 (CH), 112.0 (CH), 99.8 (CH); HRMS *m/z*: 245.0390 found (calculated for C_12_H_8_N_2_OS_2_, [M+H]^+^ requires 245.0386).

*(Z)-4-(Benzo[b]thiophen-3-ylmethylene)-2-(methylthio)-1H-imidazol-5(4H)-one* (**3a**). Ethyl iodide (2.15 g, 15.1 mmol) was added to a solution of **2a** (2.9 g, 11.1 mmol), in 15% aqueous NaOH (4.5 mL) and 95% methanol (30 mL) and the resulting reaction mixture was stirred for 4 h at room temperature. Most of the solvent was removed by distillation and to the precipitate, was added 15 mL of water and stirred for 10 min and filtered and washed first with water (200 mL) and then with diethyl ether (50 mL) to supply 2.85 g of (*Z*)-4-(benzo[*b*]thiophen-3-ylmethylene)-2-(methylthio)-1*H*-imidazol-5(4*H*)-one (**3a**) as a bright yellow solid. m.p.: 276–278 °C; IR (KBr) ν (cm^−1^): 3058, 1701, 1627, 1555, 1492; ^1^H-NMR (DMSO-*d_6_*) δ: 11.84 (brs, 1H, NH), 8.94 (s, 1H, Ar–CH), 8.11 (d, *J* = 7.5 Hz, 1H, Ar–CH), 8.01 (d, *J* = 8Hz, 1H, Ar–CH), 7.44–7.40 (m, 2H, Ar–CH), 7.04 (s, 1H, vinylic proton), 2.66 (s, 3H, SCH_3_); ^13^C-NMR (DMSO-*d_6_*) δ: 170.9 (C), 165.9 (C), 140.2 (C), 139.4 (C), 138.5 (C), 133.0 (CH), 130.1 (C), 125.4 (CH), 123.5 CH), 121.9 (CH), 111.2 (CH), 12.9 (SCH_3_); HRMS *m/z*: 275.0322 found (calculated for C_13_H_10_N_2_OS_2_, [M+H]^+^ requires 275.0315).

*(Z)-4-(Benzo[b]thiophen-3-ylmethylene)-1-methyl-2-(methylthio)-1H-imidazol-5(4H)-one* (**3b**). Compound **3b** was prepared in same manner as **3a** with the exception that an excess (2.5 equiv.) of methyl iodide was used and stirring was carried out for 16 h. This reaction afforded 2.9 g (90% yield) of **3b** as a light brown solid. m.p.: 260–263 °C; IR (KBr) ν (cm^−1^): 1700, 1643, 1540, 1497; ^1^H-NMR (DMSO-*d_6_*) δ: 8.99 (s, 1H, Ar–CH), 8.16 (d, *J* = 7.5 Hz, 1H, Ar–CH), 8.03 (d, *J* = 7.5 Hz, 1H, Ar–CH), 7.46–7.41 (m, 2H, Ar–CH), 7.14 (s, 1H, vinylic proton), 3.06 (s, 3H, NCH_3_), 2.72 (s, 3H, SCH_3_); ^13^C-NMR (DMSO-*d_6_*) δ: 169.1 (C), 166.9 (C), 139.4 (C), 139.1 (C), 138.5 (C), 133.6 (CH), 130.0 C), 125.5 (CH), 123.5 (CH), 122.1 (CH), 112.4 (CH), 26.9 (CH_3_), 13.2 (CH_3_); HRMS *m/z*: 289.0483 found (calculated for C_14_H_12_N_2_OS_2_, [M+H]^+^ requires 289.0471).

*(Z)-4-(Benzofuran-3-ylmethylene)-1-methyl-2-(methylthio)-1H-imidazol-5(4H)-one* (**3c**). Compound **3c** was obtained as a brown solid in 89% when prepared by the same method used for the preparation of **3a**. m.p.: 168–171 °C; IR (KBr) 1701, 1642, 1541, 1496 cm^−1^; ^1^H NMR (DMSO-*d_6_*): δ 8.73 (s, 1H, Ar–CH), 8.29 (d, *J* = 7.45 Hz, 1H, Ar–CH), 7.60 (d, *J* = 7.45 Hz, 1H, Ar–CH, 7.35–7.29 (m, 2H, Ar–CH), 7.05 (s, 1H, vinylic proton), 3.03 (s, 3H, NCH_3_), 2.70 (s, 3H, SCH_3_); ^13^C-NMR (DMSO-*d_6_*) δ: 168.5 (C), 165.7 (C), 155.2 (C), 150.4 (CH), 138.7 (C), 126.1 (C), 125.8 (CH), 124.0 (CH), 121.9 (CH), 117.0 (C), 112.3 (CH), 112.0 (CH), 26.8 (CH_3_), 13.2 (CH_3_); HRMS *m/z*: 273.0701 found (calculated for C_14_H_12_N_2_O_2_S, [M+H]^+^ requires 273.0699).

*(Z)-4-(Benzo[b]thiophen-3-ylmethylene)-2-(piperidin-1-yl)-1H-imidazol-5(4H)-one* (**5a**). Compound **3a** and piperidine (excess, 12.5 equiv.) were mixed then placed in a specially designed MW test tube. Ethanol (2 mL) was added to the mixture. The test tube was then sealed and then irradiated for 30 min at 100 °C and 150 psi pressure. After cooling, the solid mass was filtered and crashed into 20 mL of 95% ethanol. The solid mass collected was washed with ethanol-hexane mixture (1:5 v/v) and dried over vacuum to get the desired product. The titled compound was obtained as bright yellow solid. m.p.: 275–277 °C; IR (KBr) ν (cm^−1^): 3111.7, 1695, 1643, 1588; ^1^H-NMR (DMSO-*d_6_*) δ: 11.20 (brs, 1H, NH), 8.67 (s, 1H, Ar–CH), 7.98 (dd, *J* = 4.5 Hz, 7.4 Hz, 2H, Ar–CH), 7.42–7.37 (m, 2H, Ar–CH), 6.63 (s, 1H, vinylic proton), 3.59 (brs, 4H, CH_2_ × 2), 1.58-1.55 (m, 6H, CH_2_ × 3); ^13^C-NMR (DMSO-*d_6_*) δ: 172.5 (C), 159.3 (C), 139.4 (C), 138.7 (C), 131.4 (C), 128.0 (CH), 125.1 (CH), 125.0 (CH), 123.4 (CH), 121.7 (CH), 101.5(CH), 45.0 (NCH_2_), 25.7 (CH_2_), 24.1 (CH_2_); HRMS *m/z*: 312.1173 found (calculated for C_17_H_17_N_3_OS, [M+H]^+^ requires 312.1165) All the compounds **5b–n** were prepared in the same way.

*(Z)-4-(Benzo[b]thiophen-3-ylmethylene)-2-morpholino-1H-imidazol-5(4H)-one* (**5b**). This compound was obtained as light yellow solid. m.p.: 292–294 °C; IR (KBr) ν (cm^−1^): 3140, 1686, 1648, 1591, 1423; ^1^H-NMR (DMSO-*d_6_*) δ: 11.29 (brs, 1H, NH), 8.72 (s, 1H, Ar–CH), 7.99 (dd, *J* = 4.5 Hz, 7.6 Hz, 2H, Ar–CH), 7.42–7.37 (m, 2H, Ar–CH), 6.69 (s, 1H, vinylic proton), 3.67–3.62 (m, 8H, CH_2_ × 4); ^13^C-NMR (DMSO-*d_6_*) δ: 172.2 (C), 159.4 (C), 139.4 (C), 138.7 (C), 131.2 (C), 128.5 (CH), 125.2 (CH), 123.5 (CH), 121.8 (CH), 102.7 (CH), 66.1 (OCH_2_), 40.3 (NCH_2_); HRMS *m/z*: 314.0963 found (calculated for C_16_H_15_N_3_O_2_S, [M+H]^+^ requires 314.0957).

*(Z)-4-(Benzo[b]thiophen-3-ylmethylene)-2-(3-methylpiperidin-1-yl)-1H-imidazol-5(4H)-one* (**5c**). This compound was obtained as white solid. m.p.: 242–244 °C; IR (KBr) ν (cm^−1^): 3206, 1692, 1616, 1426, 1407; ^1^H-NMR (DMSO-*d_6_*) δ: 11.15 (brs, 1H, NH), 8.66 (s, 1H, Ar–CH), 7.99 (dd, *J* = 4.3 Hz, 7.9 Hz, 2H, Ar–CH), 7.42–7.37 ( m, 2H, Ar–CH), 6.62 (s, 1H, vinylic proton), 4.25 (brs, 2H, CH_2_), 3.01 (dd, *J* = 10 Hz, 10.5 Hz, 1H, CH_2_), 2.70 (brs, 1H, CH_2_), 1.76–1.44 (m, 4H, CH_2_ × 2), 1.17–1.13 (m, 1H, CH), 0.87 (d, *J* = 4.5 Hz, 3H, CH_3_); ^13^C-NMR (DMSO-*d_6_*) δ: 172.3 (C), 159.5 (C), 139.4 (C), 138.7 (C), 131.4 (C), 127.9 (CH), 125.1 (CH), 125.0 (CH), 123.4 CH), 121.7 (CH), 101.4 (CH), 51.2 (NCH_2_), 45.3 (NCH_2_), 32.6 (CH_2_), 31.0 (CH), 24.9 (CH_2_), 18.9 (CH_3_); HRMS *m/z*: 326.1334 found (calculated for C_18_H_19_N_3_OS, [M+H]^+^ requires 326.1322).

*(Z)-4-(Benzo[b]thiophen-3-ylmethylene)-1-methyl-2-(piperidin-1-yl)-1H-imidazol-5(4H)-one* (**5d**). This compound was obtained as light yellow solid. m.p.: 165–168 °C; IR (KBr) ν (cm^−1^): 1713, 1638, 1560, 1451; ^1^H-NMR (DMSO-*d_6_*) δ: 8.77 (s, 1H, Ar-CH), 8.02 (dd, *J* = 7.2 Hz, 7.8 Hz, 2H, Ar–CH), 7.43–7.38 (m, 2H, Ar–CH), 6.84 (s, 1H, vinylic proton), 3.55 (s, 4H, CH_2_ × 2), 3.17 (s, 3H, NCH_3_), 1.62 (s, 6H, CH_2_ × 3); ^13^C-NMR (DMSO-*d_6_*) δ: 171.5 (C), 161.7 (C), 140.3 (C), 139.4 (C), 138.6 (C), 131.1 CH), 129.5 (CH), 125.2 (CH), 125.1 (CH), 123.5 (CH), 121.9 (CH), 105.3 (CH), 48.3 (NCH_2_), 30.4 (NCH_3_), 25.6 (CH_2_), 24.2 (CH_2_); HRMS *m/z*: 326.1335 found (calculated for C_18_H_19_N_3_OS, [M+H]^+^ requires 326.1322).

*(Z)-4-(Benzo[b]thiophen-3-ylmethylene)-2-(3,5-dimethylmorpholino)-1H-imidazol-5(4H)-one* (**5e**). This compound was obtained as bright yellow solid. m.p.: 240–242 °C. IR (KBr) ν (cm^−1^): 3112, 1696, 1640, 1584, 1452; ^1^H-NMR (DMSO-*d_6_*) δ: 11.30 (brs, 1H, NH), 8.74 (s, 1H, Ar-CH), 7.99 (dd, *J* = 4.1 Hz, 7.8 Hz, 2H, Ar–CH), 7.42–7.37 (m, 2H, Ar–CH), 6.66 (s, 1H, vinylic proton). 4.01–3.98 (m, 2H, CH), 3.61–3.60 (m, 2H, CH_2_), 2.71 (brs, 2H, CH_2_), 1.13–1.11 (m, 6H, CH_3_ × 2); ^13^C-NMR (DMSO-*d_6_*) δ: 172.0 (C), 158.8 (C), 142.3 (C), 139.4 (C), 138.7 (C), 131.2 CH), 128.7 (CH), 125.1 (CH), 123.5 (CH), 121.7 (CH), 102.7 (CH), 71.3 (OCH_3_), 60.1 (CH) 19.0 (CH_3_); HRMS *m/z*: 342.1282 found (calculated for C_18_H_19_N_3_O_2_S, [M+H]^+^ requires 342.1271).

*(Z)-4-(Benzo[b]thiophen-3-ylmethylene)-1-methyl-2-morpholino-1H-imidazol-5(4H)-one* (**5f**). This compound was obtained as bright yellow solid. m.p.: 154–156 °C. IR (KBr) ν (cm^−1^): 1717, 1641, 1561, 1465; ^1^H-NMR (DMSO-*d_6_*) δ: 8.79 (s, 1H, Ar–CH), 8.05 (dd, *J* = 7.45 Hz, 7.7 Hz, 2H, Ar–CH), 7.43–7.38 (m, 2H, Ar–CH), 6.89 (s, 1H, vinylic proton), 3.72–3.70 (m, 4H, CH_2_ × 2), 3.62–3.60 (m, 4H, CH_2_ × 2), 3.18 (s, 3H, NCH_3_); ^13^C-NMR (DMSO-*d_6_*) δ: 171.2 (C), 161.5 (C), 139.9 (C), 139.3 (C), 138.6 (C), 130.9 (CH), 130.1 (CH), 125.3 (CH), 125.2 (CH), 123.5 (CH), 121.9 (CH), 106.2 (CH), 66.1 (OCH_2_), 40.3 (NCH_2_), 30.2 (NCH_3_); HRMS *m/z*: 328.1123 found (calculated for C_17_H_17_N_3_O_2_S, [M+H]^+^ requires 328.1114).

*(Z)-4-(Benzo[b]thiophen-3-ylmethylene)-1-methyl-2-(3-methylpiperidin-1-yl)-1H-imidazol-5(4H)-one* (**5g**). This compound was obtained as light yellow solid. m.p.: 150–152 °C; IR (KBr) ν (cm^−1^): 1709, 1637, 1561, 1457, 1439; ^1^H-NMR (DMSO-*d_6_*) δ: 8.75 (s, 1H, Ar–CH), 8.05 (dd, *J* = 7.2 Hz, 7.8 Hz, 2H, Ar–CH), 7.43-7.38 (m, 2H, Ar–CH), 6.83 (s, 1H, vinylic proton), 4.01–3.97 (m, 2H, CH_2_), 3.18 (s, 3H, NCH_3_), 3.02–2.9 (m, 1H, CH), 2.73–2.70 (m, 2H, CH_2_), 1.72–1.69 (m, 4H, CH_2_ × 2), 1.17–1.13 (m, 2H, CH_2_), 0.90 (d, *J* = 6.3 Hz, 3H, CH_3_); ^13^C-NMR (DMSO-*d_6_*) δ: 171.5 (C), 161.6 (C), 140.3 (C), 139.4 (C), 138.6 (C), 131.1 (CH), 129.5 (CH), 125.3 (CH), 125.2 (CH), 123.5 (CH), 121.9 (CH), 105.2 (CH), 54.3 (CH_2_), 47.8 (CH_2_), 32.7 (CH_2_), 31.0 (CH_2_), 30.4 (CH), 25.01 (CH_3_), 19.3 (CH_3_); HRMS *m/z*: 340.1485 found (calculated for C_19_H_21_N_3_OS, [M+H]^+^ requires 340.1478).

*(Z)-4-(Benzo[b]thiophen-3-ylmethylene)-2-thiomorpholino-1H-imidazol-5(4H)-one* (**5h**). This compound was obtained as bright yellow solid. m.p.: 269–271 °C; IR (KBr) ν (cm^−1^): 3110, 1694, 1640, 1585, 1490, 1453; ^1^H-NMR (DMSO-*d_6_*) δ: 11.31 (brs, 1H, NH), 8.70 (s, 1H, Ar–CH), 8.00–7.97 (m, 2H, Ar–CH), 7.42–7.37 (m, 2H, Ar–CH), 6.67 (s, 1H, vinylic proton), 3.89 (brs, 4H, CH_2_ × 2), 2.68 (s, 4H, CH_2_ × 2); ^13^C-NMR (DMSO-*d_6_*) δ: 172.5 (C), 158.9 (C), 139.5 (C), 138.6 (C), 131.5 (C), 128.7 (CH), 125.5 (CH), 123.5 (CH), 121.3 (CH), 102.1 (CH), 46.3 (NCH_2_), 26.3 (SCH_3_); HRMS *m/z*: 330.0737 found (calculated for C_16_H_15_N_3_OS_2_, [M+H]^+^ requires 330.0729).

*(Z)-4-(Benzo[b]thiophen-3-ylmethylene)-2-(4-methylpiperazin-1-yl)-1H-imidazol-5(4H)-one* (**5i**). This compound was obtained as yellow solid. m.p.: 234–236 °C; IR (KBr) ν (cm^−1^): 3108, 1697, 1646, 1591, 1448; ^1^H-NMR (DMSO-*d_6_*) δ 11.15 (brs, 1H, NH), 8.68 (s, 1H, Ar–CH), 7.99 (dd, *J* = 4 Hz, 8 Hz, 2H, Ar–CH), 7.42–7.37 (m, 2H, Ar–CH), 6.64 (s, 1H, vinylic proton), 3.61 (brs, 4H, CH_2_ × 2), 2.37 (s, 4H, CH_2_ × 2), 2.19 (s, 3H, NCH_3_); ^13^C-NMR (DMSO-*d_6_*) δ: 172.6 (C), 159.6 (C), 139.3 (C), 138.6 (C), 131.3 (C), 128.4 (CH), 125.1 (CH), 123.5 (CH), 121.7 (CH), 102.2 (CH), 54.4 (NCH_2_), 46.0 (NCH_2_), 45.4(NCH_3_); HRMS *m/z*: 327.1290found (calculated for C_17_H_18_N_4_OS, [M+H]^+^ requires 327.1281).

*(Z)-4-(Benzofuran-3-ylmethylene)-1-methyl-2-(piperidin-1-yl)-1H-imidazol-5(4H)-one* (**5j**). This compound was obtained as brown solid. m.p.: 148–150 °C; IR (KBr) ν (cm^−1^): 1713, 1649, 1556, 1450; ^1^H-NMR (DMSO-*d_6_*) δ: 8.61 (s, 1H, Ar–CH), 8.14 (d, *J* = 7.45 Hz, 1H, Ar–CH), 7.57 (d, *J* = 7.45 Hz, 1H, Ar–CH), 7.34–7.28 (m, 2H, Ar–CH), 6.70 (s, 1H, vinylic proton), 3.53 (s, 4H, CH_2_ × 2), 3.15 (s, 3H, NCH_3_), 1.62 (s, 6H, CH_2_ × 3); ^13^C-NMR (DMSO-*d_6_*) δ: 170.8 (C), 161.0 (C), 154.9 (C), 148.0 (CH), 140.0 (C), 126.6 (C), 125.4 (CH), 123.6 (CH), 121.4 (CH), 117.4 (C), 111.9 (CH), 104.4 (CH), 48.3 (NCH_2_), 30.3 (NCH_3_), 25.6 (CH_2_), 24.2 (CH_2_); HRMS *m/z*: 310.1561 found (calculated for C_18_H_19_N_3_O_2_, [M+H]^+^ requires 310.1557).

*(Z)-4-(Benzofuran-3-ylmethylene)-1-methyl-2-morpholino-1H-imidazol-5(4H)-one* (**5k**). This compound was obtained as yellow solid. m.p.: 142–145 °C; IR (KBr) ν (cm^−1^): 1708, 1644, 1557, 1452; ^1^H-NMR (ACETONE-*d_6_*) δ: 8.62 (s, 1H, Ar–CH), 8.06 (d, *J* = 7.00 Hz, 1H, Ar–CH), 7.51 (d, *J* = 7.00 Hz, 1H, Ar–CH), 7.35–7.29 (m, 2H, Ar–CH), 6.77 (s, 1H, vinylic proton), 3.77 (s, 4H, OCH_2_ × 2), 3.60 (s, 4H, NCH_2_ × 2), 3.19 (s, 3H, NCH_3_); ^13^C-NMR (ACETONE-*d_6_*) δ: 170.1 (C), 160.9 (C), 155.0 (C), 148.0 (CH), 139.7 (C), 126.7 (C), 124.8 (CH), 123.1(CH), 120.4 (CH), 117.1 (C), 111.3 (CH), 105.3 (CH), 65.9 (OCH_2_), 47.5 (NCH_2_), 28.9 (NCH_3_); HRMS *m/z*: 312.1358 found (calculated for C_17_H_17_N_3_O_3_, [M+H]^+^ requires 312.1350).

*(Z)-4-(Benzofuran-3-ylmethylene)-1-methyl-2-thiomorpholino-1H-imidazol-5(4H)-one* (**5l**). This compound was obtained as yellowish solid. m.p.: 157–159 °C; IR (KBr) ν (cm^−1^): 1703, 1649, 1541, 1450; ^1^H-NMR (DMSO-*d_6_*) δ: 8.62 (s, 1H, Ar–CH), 8.14 (d, *J* = 7.45 Hz, 1H, Ar–CH), 7.58 (d, *J* = 7.45 Hz, 1H, Ar–CH), 7.33–7.29 (m, 2H, Ar–CH), 6.76 (s, 1H, vinylic proton), 3.83–3.81 (m, 4H, CH_2_ × 2), 3.15 (s, 3H, NCH_3_), 2.77–2.75 (m, 4H, CH_2_ × 2); ^13^C-NMR (DMSO-*d_6_*) δ: 170.6 (C), 160.9 (C), 154.9 (C), 148.3 (CH), 139.6 (C), 126.6 (C), 125.5 (CH), 123.7 (CH), 121.4 (CH), 117.3 (C), 111.9 (CH), 105.5 (CH), 49.9 (NCH_2_), 30.2 (SCH_2_), 26.5 (NCH_3_); HRMS *m/z*: 328.1132 found (calculated for C_17_H_17_N_3_O_2_S, [M+H]^+^ requires 328.1121).

*(Z)-4-(Benzofuran-3-ylmethylene)-1-methyl-2-(4-methylpiperazin-1-yl)-1H-imidazol-5(4H)-one* (**5m**). This compound was obtained as a low melting yellow solid. IR (KBr) ν (cm^−1^): 1708, 1644, 1559, 1450; ^1^H-NMR (DMSO-*d_6_* + acetone-*d_6_*) δ: 9.09 (s, 1H, Ar–CH), 8.57 (d, *J* = 7.45 Hz, 1H, Ar–CH), 8.00 (d, *J* = 7.45 Hz, 1H, Ar–CH), 7.81–7.74 (m, 2H, Ar–CH), 7.21 (s, 1H, vinylic proton), 4.07 (t, *J* = 4.6 Hz, 4H, CH_2_ × 2), 3.66 (s, 3H, CH_3_), 2.93 (t, *J* = 4.6 Hz, 4H, CH_2_ × 2), 2.68 (s, 3H, CH_3_); ^13^C-NMR (DMSO-*d_6_* + ACETONE-*d_6_*) δ: 170.9 (C), 161.4 (C), 155.4 (C), 148.5 (CH), 140.3 (C), 127.1 (C), 125.6 (CH), 123.8 (CH), 121.4 (CH), 117.7 (C), 112.0 (CH), 105.4 (CH), 54.8 (CH_2_), 47.6 (CH_2_), 46.2 (CH_3_), 30.2 (CH_3_); HRMS *m/z*: 325.1670 found (calculated for C_18_H_20_N_4_O_2_, [M+H]^+^ requires 325.1666).

*(Z)-4-(Benzofuran-3-ylmethylene)-2-(3,5-dimethylmorpholino)-1-methyl-1H-imidazol-5(4H)-one* (**5n**). This compound was obtained as bright yellow solid. m.p.: 137–140 °C; IR (KBr) ν (cm^−1^): 1703, 1641, 1557, 1456; ^1^H-NMR (DMSO-*d_6_*) δ: 8.63 (s, 1H, Ar–CH), 8.15 (d, *J* = 7.45 Hz, 1H, Ar–CH), 7.58 (d, *J* = 7.45 Hz, 1H, Ar–CH), 7.33–7.28 (m, 2H, Ar–CH), 6.74 (s, 1H, vinylic proton), 3.98 (d, *J* = 12.6 Hz, 2H, CH_2_), 3.71–3.69 (m, 2H, CH_2_), 3.18 (s, 3H, CH_3_), 2.78–2.75 (m, 2H, CH), 1.13 (d, *J* = 12.6 Hz, 6H, CH_3_ × 2); ^13^C-NMR (DMSO-d_6_) δ: 170.7 (C), 160.4 (C), 154.9 (C), 148.3 (CH), 139.6 (C), 126.6 (C), 125.8 (CH), 123.6 (CH), 121.4(CH), 117.3 (C), 112.0 (CH), 105.3 (CH), 71.2 (OCH_2_), 52.2 (CH), 30.2 (CH_3_), 19.0 (CH_3_); HRMS *m/z*: 340.1671 found (calculated for C_19_H_21_N_3_O_3_, [M+H]^+^ requires 340.1663).

## 4. Conclusions

In summary we have successfully developed a synthetic method that provides ready access to novel biologically important benzo[*b*]thiophene and benzofuran based thiohydantoin derivatives. We are currently investigating the synthesis of a number of other thiohydantoin-based drug molecules by this method. A detailed biological activity study (antibacterial, antifungal, anticancer and neuroprotective kinase inhibitor activity) of these important compounds is being carried out. Preliminary results indicate that many of the thiohydantoins exhibit excellent neuroprotective properties.
